# Quantitative molecular phenotyping with topically applied SERS nanoparticles for intraoperative guidance of breast cancer lumpectomy

**DOI:** 10.1038/srep21242

**Published:** 2016-02-16

**Authors:** Yu Wang, Soyoung Kang, Altaz Khan, Gabriel Ruttner, Steven Y. Leigh, Melissa Murray, Sanjee Abeytunge, Gary Peterson, Milind Rajadhyaksha, Suzanne Dintzis, Sara Javid, Jonathan T.C. Liu

**Affiliations:** 1 Department of Mechanical Engineering, University of Washington, Seattle, WA 98195, USA; 2 Department of Biomedical Engineering, Stony Brook University (SUNY), Stony Brook, NY 11794, USA; 3 Department of Pathology, Memorial Sloan Kettering Cancer Center, New York, NY 10065, USA; 4 Dermatology Service, Memorial Sloan Kettering Cancer Center, New York, NY 10065, USA; 5 Department of Pathology, University of Washington School of Medicine, Seattle, WA 98195, USA; 6 Department of Surgery, University of Washington School of Medicine, Seattle, WA 98195, USA

## Abstract

There is a need to image excised tissues during tumor-resection procedures in order to identify residual tumors at the margins and to guide their complete removal. The imaging of dysregulated cell-surface receptors is a potential means of identifying the presence of diseases with high sensitivity and specificity. However, due to heterogeneities in the expression of protein biomarkers in tumors, molecular-imaging technologies should ideally be capable of visualizing a multiplexed panel of cancer biomarkers. Here, we demonstrate that the topical application and quantification of a multiplexed cocktail of receptor-targeted surface-enhanced Raman scattering (SERS) nanoparticles (NPs) enables rapid quantitative molecular phenotyping (QMP) of the surface of freshly excised tissues to determine the presence of disease. In order to mitigate the ambiguity due to nonspecific sources of contrast such as off-target binding or uneven delivery, a ratiometric method is employed to quantify the specific vs. nonspecific binding of the multiplexed NPs. Validation experiments with human tumor cell lines, fresh human tumor xenografts in mice, and fresh human breast specimens demonstrate that QMP imaging of excised tissues agrees with flow cytometry and immunohistochemistry, and that this technique may be achieved in less than 15 minutes for potential intraoperative use in guiding breast-conserving surgeries.

Breast-conserving surgery, or lumpectomy, has superseded mastectomy as the most common surgical method for the removal of breast tumors, comprising 70% of surgeries for early-stage breast cancer. Multiple randomized controlled clinical trials have demonstrated similar survival outcomes and low local-recurrence rates for both lumpectomy and mastectomy^1^,^2^. Unfortunately, amongst various clinical institutions, anywhere from 20–60% of lumpectomy patients must undergo additional surgery when post-operative pathology reveals tumor at or near the surgical margins, indicating an incomplete removal of the tumor mass^3^,^4^. Such re-excision surgeries are costly, inconvenient for patients, increase the risk of iatrogenic injury, and may result in delayed adjuvant therapies with inferior patient outcomes.

There is controversy amongst breast-cancer oncologists over the criteria that warrant re-excision following initial lumpectomy. Many institutions advocate re-excision procedures for close margins, in which tumor is found within a certain distance (often 2 to 3 mm) of the surgical margin, particularly for ductal carcinoma *in situ*. However, a national consensus that defined a negative margin as “no tumor at the inked margin” (*i.e.* no tumor at the surface of the surgical excision) for invasive breast cancer was reached in 2014^5^. Regardless of one’s position on these issues, there is little debate over the necessity for re-excision surgeries when a lumpectomy margin is positive for tumor (tumor at the surface of the final excised specimen, *i.e.* “tumor on ink”). Intraoperative specimen X-ray imaging of lumpectomy specimens helps guide the surgeon in detecting grossly positive margins (i.e. calcifications or tumor present at the radiographic margins). However, specimen X-ray (as with mammography) is diagnostically inaccurate, and there are currently no tools to enable surgeons to detect positive margins with high sensitivity and specificity. In particular, frozen-section pathology, which is often utilized to guide the resection of other tumor types, is difficult to perform for breast tissues, and often inaccurate due to their high lipid content. Furthermore, post-operative pathology of formalin-fixed breast tissues, though microscopically precise and highly accurate for identifying tumors, suffers from sampling errors due to the impracticality of micron-level sectioning and microscopic interrogation of entire resection margins^6^,^7^. There is therefore a need for an intraoperative technique to accurately identify residual tumors at the margins of freshly resected tissues to reduce the number of costly and potentially harmful re-excision surgeries for patients undergoing breast cancer lumpectomy (Fig. 1).

Molecular-imaging approaches have the potential to identify tumors with a high degree of sensitivity and specificity^8^,^9^,^10^,^11^. However, a confounding factor is that disease biomarkers vary greatly between patients, within a tumor over time, as well as at different locations within a tumor mass^12^,^13^. Therefore, exogenous probes should ideally be capable of being multiplexed to image a diverse panel of disease-related biomarkers simultaneously and to determine a “quantitative molecular phenotype (QMP)” of the tissue under consideration. In recent years, surface-enhanced Raman-scattering (SERS) nanoparticles (NPs), hereafter referred to as “SERS NPs” or “NPs”, have attracted interest due to their brightness, photostability, and especially their multiplexing capability with laser illumination at a single wavelength^14^. The SERS NPs utilized in this study exist as various “flavors,” each of which generates a characteristic spectral “fingerprint” or “barcode” that uniquely identifies that NP flavor (Fig. 2d). By targeting various flavors of SERS NPs to different biomarkers and applying them simultaneously on tissues, multiplexed molecular imaging is possible in which mixed SERS spectra are detected and computationally demultiplexed to determine the relative concentrations of the individual SERS NP flavors within a mixture^15^,^16^,^17^. The QMP technology described here is a surface-imaging approach that can potentially identify residual tumors at the surgical margins, for which there is an unequivocal need for additional resection. While this large-area imaging approach lacks the cellular-level resolution of microscopic pathology, it offers the potential for comprehensive imaging of large tissue areas with sub-millimeter resolution during surgery without the sampling errors inherent to conventional post-operative pathology.

A number of groups have begun to explore the potential of SERS NPs for tumor detection in tissues^14^,^15^,^18^,^19^. A few studies have explored the behavior of SERS NPs in tumor-bearing mice after intravenous injection and imaging with a Raman microscope system^18^,^19^ whereas others have demonstrated the multiplexed detection of large panels of nontargeted SERS NPs in living mice^20^,^21^,^22^,^23^,^24^. The relatively large size of SERS NPs (20–120 nm) hampers their extravasation and penetration into most tissues when delivered systemically. While this feature has been utilized for the imaging of tumors through enhanced permeability and retention (EPR) mechanisms^25^,^26^, these passive delivery effects complicate molecular-imaging efforts and can lead to a high degree of ambiguity when one wishes to determine if image contrast is due to true molecular binding rather than passive accumulation. Alternatively, the topical application of SERS NPs has been proposed as a means of labeling disease biomarkers that are located on exposed surfaces such as epithelial cancers or surgically exposed/resected tissues. For example, one study demonstrated the feasibility of detecting a single biomarker after topically staining *ex vivo* tissues from mice for 1 h with targeted SERS-NPs^27^. More recently, we demonstrated that multiplexed SERS NPs could be topically applied for just 5 min on fresh tumor xenografts in mice, followed by a rapid rinse-removal step (20 s), to enable the simultaneous quantification of multiple biomarkers using a single-point contact probe^17^. Topical delivery of SERS NPs on *ex vivo* resected tissues is attractive because it circumvents toxicity issues and can potentially expedite the regulatory-approval process compared with contrast agents that are administered systemically.

In the study described here, we demonstrate the feasibility of utilizing SERS NPs to enable quantitative multiplexed molecular imaging of freshly excised human tissues for surgical-guidance applications. A topical-application protocol was first optimized to maximize the molecular image contrast between tumor and normal tissues. Previously we demonstrated that the manual positioning of a contact Raman probe (a single-point measurement device) enabled the identification of tumor xenografts topically stained with targeted SERS NPs^17^. Here, by integrating a spatially offset Raman probe with a 2D-raster-scanning platform, large tissue areas could be spectrally imaged with tunable spatial resolution (0.2–1 mm). We further demonstrate that the staining and QMP imaging of fresh tissue surfaces (approx. 4-cm^2^) can be achieved within 15 minutes and that the QMP images agree with flow cytometry and immunohistochemistry (IHC) validation data. Experiments with human breast tissues demonstrate that this technique can achieve rapid comprehensive imaging of biopsy shavings to potentially guide the final stages of breast-conserving surgeries.

A critical component of our imaging technique, which eliminates the ambiguities due to nonspecific sources of contrast, is the use of a quantitative ratiometric-imaging method (Fig. 1). As an example to motivate the need for this strategy, this study and previous studies have shown that increased diffusion and passive retention can occasionally result in higher accumulated concentrations of topically applied NPs in normal tissues in comparison to denser tumor tissues, i.e. inverse contrast^17^. Other sources of misleading nonspecific contrast include off-target chemical binding, variations in detector working distance and illumination power, as well as uneven NP delivery and removal. However, by simultaneously delivering one nontargeted NP flavor to control for the nonspecific behavior of one or more biomarker-targeted NP flavors, a calibrated ratiometric image of specific vs. nonspecific binding can be generated to identify elevated molecular expression for the purposes of differentiating between tumors and normal tissues^24^,^26^,^28^,^29^,^30^,^31^. Note that SERS NPs are particularly well-suited for ratiometric detection due to their excellent multiplexing capabilities, the identical geometry of all NP flavors, and the fact that they can be excited at a single illumination wavelength, ensuring that all NP flavors are interrogated identically in terms of optical irradiance and penetration depth (see Methods and Supplementary information for additional details). In contrast, fluorescent reporters imaged at disparate excitation and emission wavelengths often must account for variations in tissue optical properties as a function of wavelength^8^,^32^,^33^.

## Results

### Optimization of a topical-application protocol

**Figure 2:**
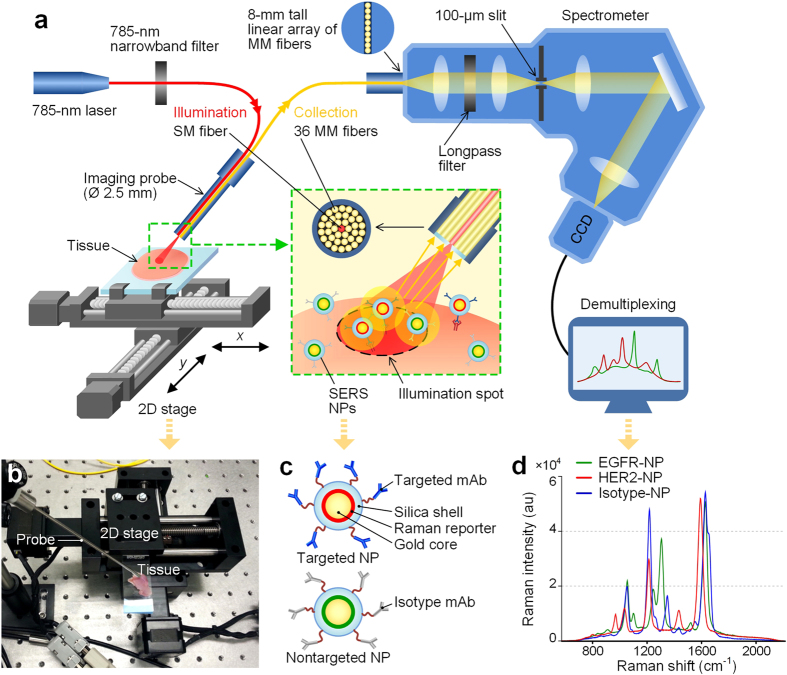
Raman imaging system. (**a**) Schematic of the spectral-imaging system. A 785-nm laser is used to illuminate the NP-stained tissue, creating a submillimeter-diameter laser spot. Raman-scattered photons from illuminated NPs are collected by 36 multimode fibers and transmitted to a customized spectrometer (Andor Holospec), where they are dispersed onto a cooled deep-depletion spectroscopic CCD (Andor). For raster-scanning imaging, a two-axis stage is controlled through a custom LabVIEW program to translate the tissue sample. (**b**) A photograph of the raster-scanned tissue-imaging device. (**c**) A depiction of the structure of the targeted and nontargeted SERS NPs and (**d**) the Raman spectra of the various SERS NPs (targeted and nontargeted) used in this study. Y.W. drew the figure.

**Figure 3:**
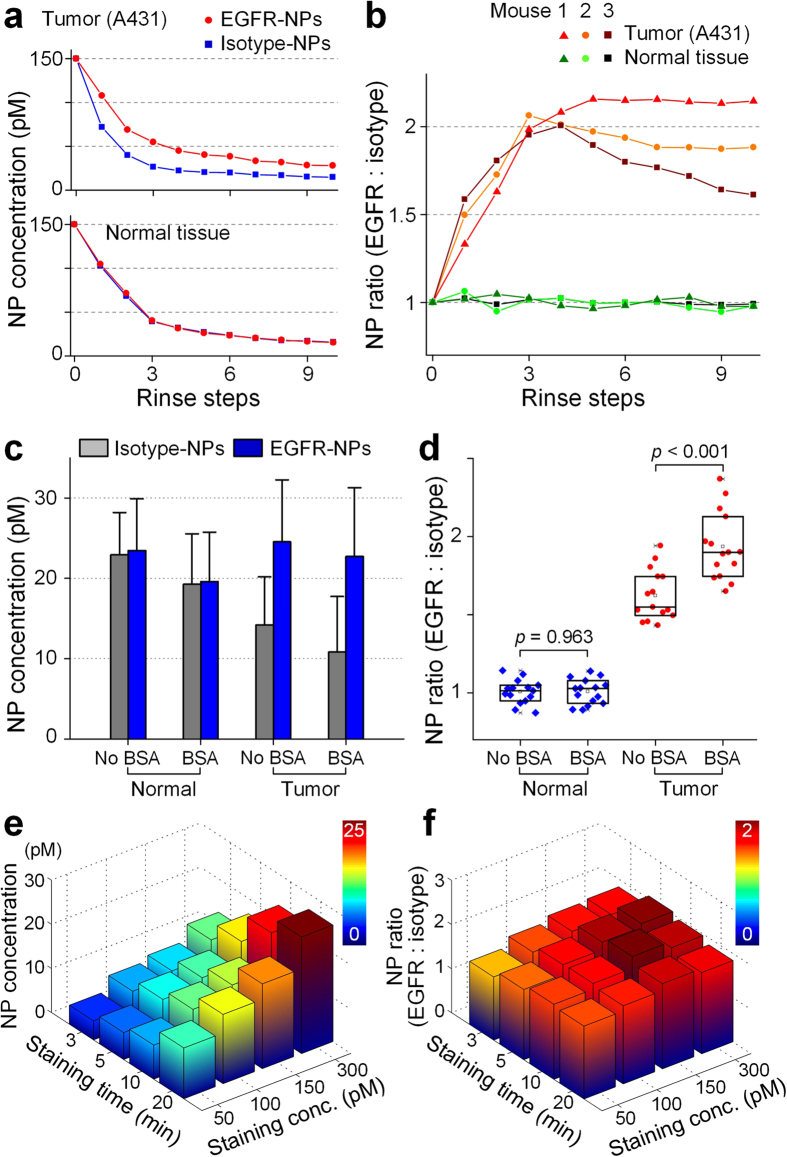
Optimization of a topical-staining procedure with tumor xenografts. (**a,b**) Multi-stage rinsing of tissue samples after they have been stained for 10 min with a 1:1 mixture of EGFR-NPs and isotype-NPs (150 pM/flavor). (**a**) Measured NP concentrations on tumor and normal tissue. (**b**) Targeted vs. nontargeted NP ratios for 3 tumors and 3 normal samples. (**c,d**) Comparison of staining efficiency with and without 1% BSA in the staining solution (150 pM per NP flavor, 10 min staining). (**c**) Measured NP concentrations. (**d**) Concentration ratio of targeted vs. nontargeted NPs. (**e**) NP concentration on A431 tumors as a function of staining duration and staining concentration. (**f**) Targeted vs. nontargeted NP ratio as a function of staining duration and staining concentration.

A custom spectral-imaging system was utilized to visualize the retention of SERS NPs topically applied on tissues (Fig. 2a). Linearity tests demonstrate the accuracy of the system for quantitatively imaging SERS NPs within a concentration range of 1–400 pM (Supplementary Fig. S1). A multi-stage rinsing method (see Methods) was first utilized to optimize the staining and rinsing conditions on tumor xenografts in order to generate maximum tumor-to-normal contrast. A 1 by 1-cm tissue sample (either an EGFR-positive tumor xenograft or EGFR-negative normal mouse tissue) was placed on a glass slide. The tissue was stained with a 10-μL NP mixture (equimolar ratio of EGFR-targeted NPs and isotype-control NPs), followed by 10 sequential rinse-and-image steps (see Methods). Progressive washout of topically applied NPs led to changes in the absolute concentrations and the concentration ratio of targeted vs. nontargeted NPs (Fig. 3a,b). On EGFR-positive tumor xenograft specimens, the concentration ratio of EGFR-NPs vs. isotype-NPs rapidly increased to ~2.0 after three rinse steps (Fig. 3b), whereas the concentration ratio remained at unity for normal (EGFR-negative) tissues. There was some variability in the results between different tissue specimens (Fig. 3b), possibly due to structural heterogeneities and varying degrees of necrosis.

Figure 3c,d show four experimental conditions (normal-noBSA, normal-BSA, tumor-noBSA, and tumor-BSA) each of which utilized 5 tissue specimens that were imaged at three locations (regions of interest) for a total of 15 data points per dataset. In these experiments, the use of BSA was explored as a blocking agent to reduce nonspecific binding^34^. The addition of 1% BSA to the staining solution reduced the non-specific accumulation of NPs within tissues, as is evidenced by the significant increase in the specific to nonspecific uptake ratio (EGFR-NP/isotype-NP) seen in Fig. 3d. Therefore, 1% BSA was employed as a nonspecific blocking agent in all subsequent experiments.

Staining concentrations and durations were optimized empirically using a matrix of conditions (Fig. 3e,f). While the absolute NP concentrations increased monotonically with increased staining concentration and staining duration (Fig. 3e), the concentration ratio of targeted vs. nontargeted NPs reached a maximum at a 150-pM staining concentration (per NP flavor) and a 10-min staining time (Fig. 3f). We hypothesize that nonspecific binding of the NPs continues to increase while specific binding begins to plateau at higher staining concentrations and longer staining times. In addition, longer staining durations may result in increased diffusion and irreversible trapping of the NPs in the tissues (or cellular internalization). Therefore, there appears to be both an optimal staining concentration and an optimal staining duration for maximizing the specific vs. nonspecific accumulation of NPs that are topically applied on tissues.

### QMP of tumor xenografts

**Figure 4:**
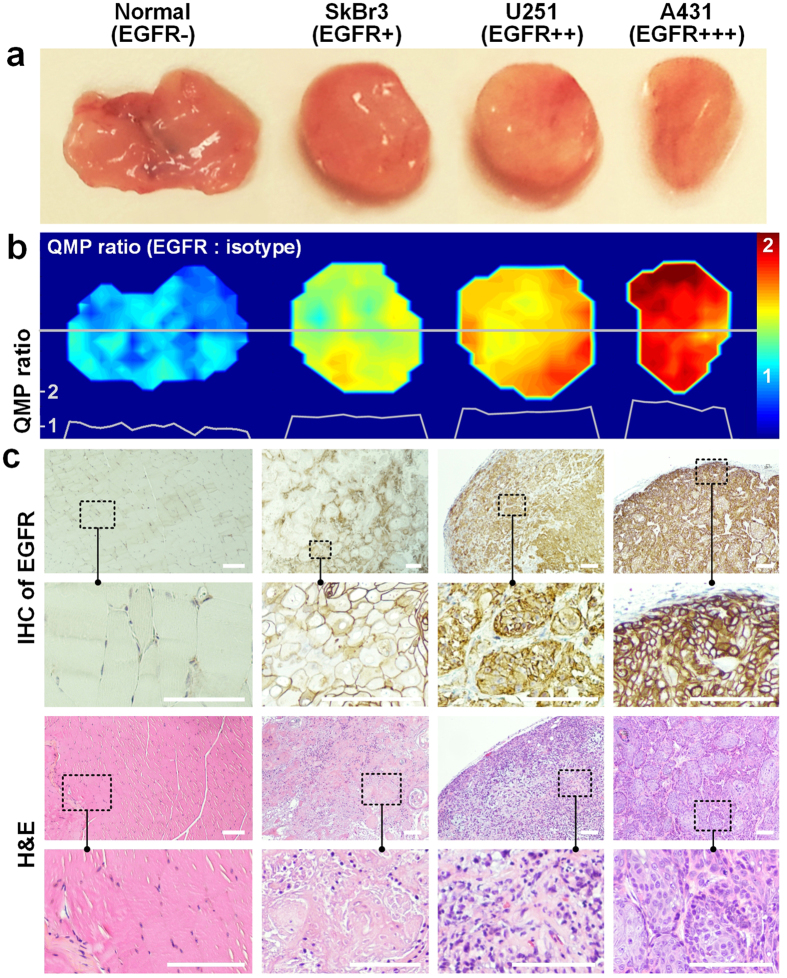
QMP imaging of normal tissue (EGFR-negative) and tumor xenografts (SkBr3, U251, and A431) that express various levels of EGFR. The tissues were stained with a two-flavor NP mixture (EGFR-NPs and isotype-NPs) and the staining-and-imaging procedure was achieved in less than 15 min. (**a**) Photographs of resected normal tissue (muscle) and tumor xenografts. (**b**) QMP images of the concentration ratio of EGFR-NPs vs. isotype-NPs. The line profiles at the bottom of the image indicate the QMP ratios along the gray line through the center of each tissue specimen. (**c**) Validation data: IHC for EGFR (10X and 40X views), and H&E staining (10X and 40X views). The scale bars represent 100 μm.

By utilizing a custom raster-scanned imaging system, we demonstrated the ability to image and accurately quantify relative biomarker expression levels in fresh tissues with topically applied SERS NPs (Fig. 2, see Methods for details). An imaging rate of 2 mm/s was utilized with an illumination spot size (resolution) of 0.5 mm and a sampling pitch of 0.5 mm/pixel. To validate this system, we first imaged tumor xenografts that were stained with an equimolar mixture of EGFR-NPs and isotype-NPs. As shown in Fig. 4b,c, the ratiometric image provides a quantitative representation of EGFR expression that is consistent with IHC and flow cytometry results (Fig. 4c, Supplementary Fig. S2). IHC results with an isotype-control (negative-control) antibody are provided in the Supplementary Fig. S4.

**Figure 5:**
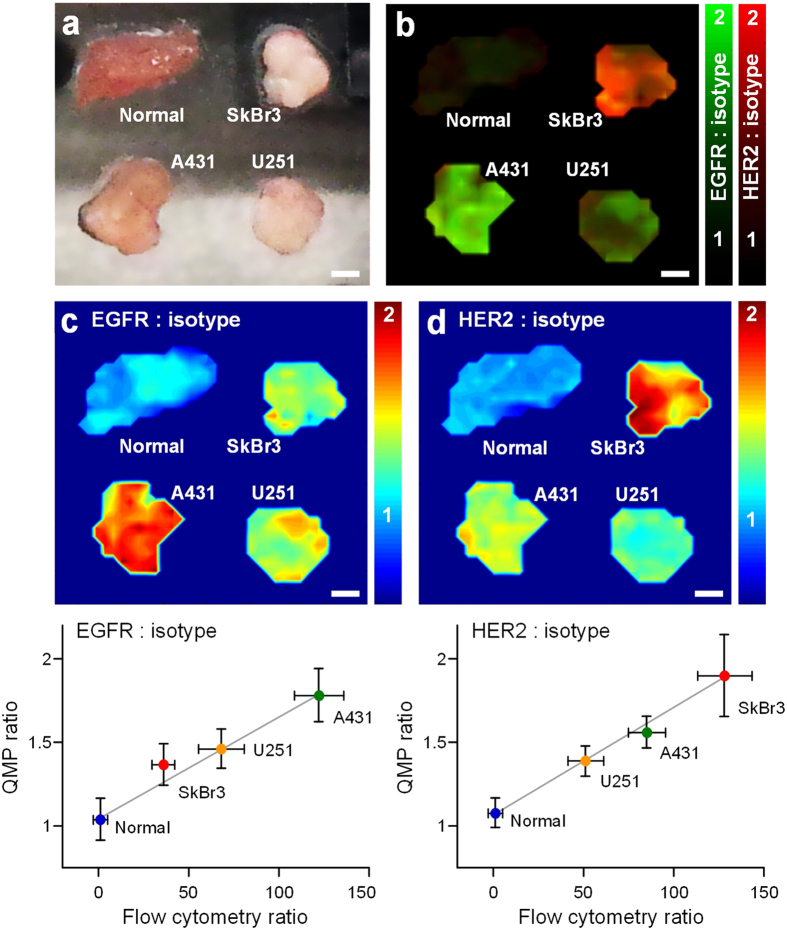
QMP imaging, with 0.5-mm spatial resolution, of tumor-xenograft specimens stained with a three-flavor NP mixture (EGFR-NPs, HER2-NPs and isotype-NPs). (**a**) Photograph of resected tumor xenografts and normal tissue. (**b**) A multiplexed QMP image generated by overlaying the ratiometric images of EGFR-NPs/isotype-NPs (plotted with a green colormap) and HER2-NPs/isotype-NPs (plotted with a red colormap). Images showing the concentration ratio of (**c**) EGFR-NPs/isotype-NPs and (**d**) HER2-NPs/isotype-NPs. The bottom plots show the correlation between the QMP ratio of a particular tissue specimen (in **c**,**d**) and the corresponding fluorescence ratio (targeted NP vs. isotype NP) from flow-cytometry experiments with the cell lines used to generate the various tumor xenografts (Supplementary Fig. S2). R > 0.98. Scale bars represent 2 mm.

To demonstrate the ability to image multiple biomarkers simultaneously, we stained various tumor xenografts with an equimolar mixture of three NP flavors - EGFR-NPs, HER2-NPs and isotype-NPs (Fig. 5). Previous flow-cytometry experiments demonstrated the high binding affinity of our targeted NPs to the cell lines that were used to generate tumor xenografts in this study (see Supplementary Fig. S2). As shown in Fig. 5b–d, the QMP images of EGFR and HER2 expression in various tumor xenografts show excellent quantitative agreement with corresponding flow-cytometry results (R > 0.98).

### QMP of human breast tissues

**Figure 6:**
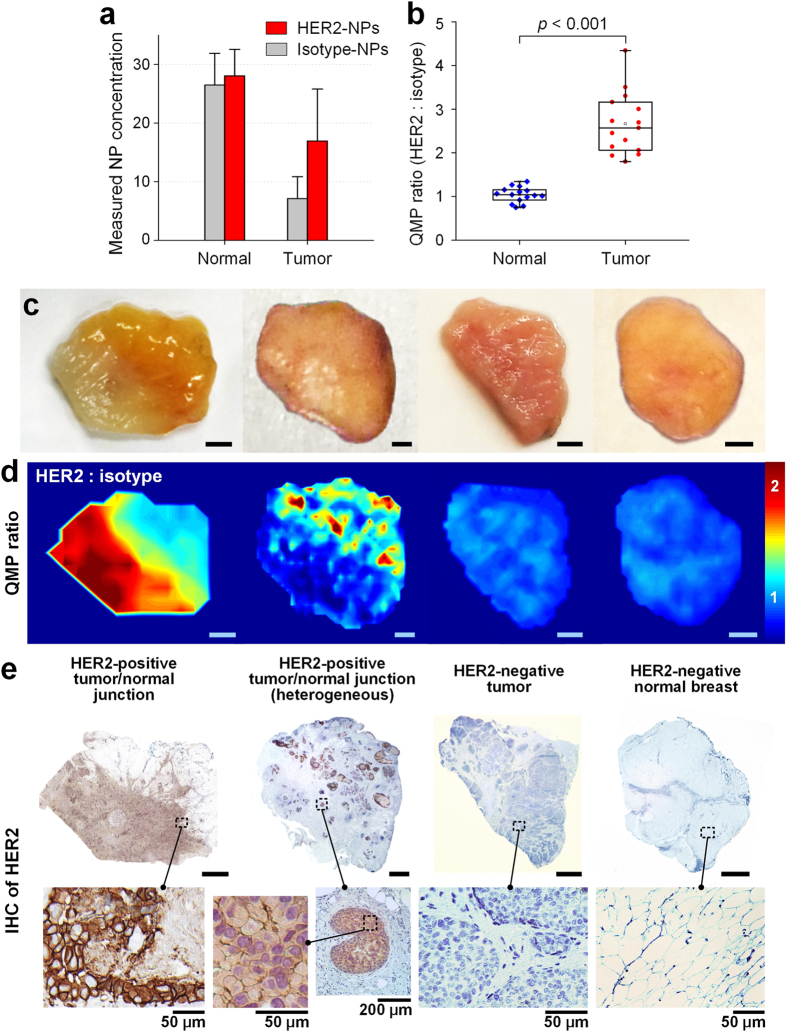
QMP imaging of human breast tissues stained with a 2-flavor NP mixture (HER2-NPs and isotype-NPs, 150 pM/flavor). (**a**) Absolute NP concentrations and (**b**) NP concentration ratios on normal tissues and tumors (10 tissue specimens from 5 patients). (**c**) Photographs of four tissue specimens from four patients: two HER2-positive specimens containing both tumor and normal tissue regions and two HER2-negative specimens (one tumor and one normal tissue). (**d**) Images of the concentration ratio of HER2-NPs vs. isotype-NPs and (**e**) IHC staining with an anti-HER2 mAb. Unlabeled scale bars represent 2 mm.

To further demonstrate the feasibility of the ratiometric QMP imaging technique for intraoperative assessment of surgical margins, we imaged 10 fresh human breast tissue specimens resected from five patients (Fig. 6). Each tissue specimen was stained with an equimolar mixture of HER2-NPs and isotype-NPs for 10 min, followed by a 20-s rinse step in PBS. An area of up to 2 × 2 cm^2^ was raster-scanned within 2 min. The entire staining-and-imaging procedure was performed in less than 15 min, a time frame that is consistent with current intraoperative guidance techniques such as 2D specimen X-ray and frozen-section pathology (not typically done for breast tissues due to their high lipid content). The concentration ratio of HER2-NPs vs. isotype-NPs measured from 10 tissue specimens ranged from 1.7 to 4.3 (Fig. 6b), which is higher than the ratio of 1.4 to 2.5 measured in HER2-positive tumor xenografts (Fig. 5d). This higher level of HER2 expression is consistent with published reports showing that HER2 expression in human breast tumors can reach 2 × 10^6^ receptors/cell or higher^35^, as compared to ~1 × 10^6^ receptors/cell in SkBr3 cells^36^. QMP images from 4 different patient specimens are shown in Fig. 6d, including a tumor-to-normal tissue junction, a sparse and spatially heterogeneous tumor junction, a HER2-negative tumor, and a normal (HER2-negative) tissue specimen. As with tumor xenografts in mice (Fig. 3c and our recent publication^17^), measurements of absolute NP concentrations are misleading: the NPs accumulate more (nonspecifically) on normal tissue regions than on the tumor regions (Fig. 6a and Supplementary Fig. S6) because the tumor xenografts and human breast tumors are denser and less porous than the surrounding normal tissues. These differences in passive delivery and nonspecific retention are a significant problem for the accurate interpretation of molecular images when only a single targeted contrast agent is used. However, ratiometric imaging of a targeted vs. untargeted NP provides accurate quantification of specific vs. nonspecific NP accumulation, and provides a measure of relative biomarker-expression levels that is in agreement with IHC validation data (Fig. 6e). Recently, we have also investigated compartmental modeling approaches to extract accurate measurements of receptor binding potential (proportional to receptor density) based on kinetic paired-agent imaging data^37^. Results of competitive-binding experiments provide additional confirmation that the targeted NPs are binding specifically to HER2 receptors in tissues (Supplementary Fig. S7). Note that in these preliminary studies, QMP imaging exhibits the sensitivity to identify submillimeter regions of HER2-positive tumor, as shown in the second HER2-positive specimen in Fig. 6d.

## Discussion

Through a series of imaging experiments with fresh tumor xenografts and fresh human breast tissues, we have demonstrated that the topical application and quantification of receptor-targeted (and non-targeted) SERS NPs allows for the rapid (<15 min) quantitative molecular phenotyping (QMP) of tissues to potentially enable the intraoperative detection of residual carcinoma at lumpectomy margins. Several features make this rapid QMP technique accurate and sensitive for the intraoperative examination of resected tissues, in excellent agreement with flow-cytometry and IHC validation data: (i) Multiplexed SERS NPs may be excited at a single illumination wavelength (785 nm), ensuring that all NP reporters in a measurement are interrogated identically in terms of illumination intensity, detection area, and effective excitation depth. This allows for the robust ratiometric quantification of specific vs. nonspecific accumulation of targeted NPs that is insensitive to off-target binding effects, the uneven topical delivery of NPs, and variations in working distance and tissue geometry. (ii) The relatively large size of these NPs (~120 nm) allows them to remain at the tissue surface rather than diffusing extensively into the tissue and being trapped (Supplementary Fig. S3), such that high molecular image contrast between tumor and normal tissues may be rapidly achieved (<15 min) with an optimized staining and rinsing procedure.

While the QMP technique is a surface-imaging method, unlike conventional pathology (which provides depth information), QMP enables the rapid intraoperative assessment of large tissue surfaces without the sampling errors that are inevitable for conventional post-operative pathology. For example, post-operative histology sections are typically cut at 5- to 10-mm intervals on lumpectomy specimens that are manually “bread loafed.” Therefore, the sub-millimeter resolution of our QMP imaging technique greatly surpasses the sampling interval of “gold-standard” histopathology. In terms of clinical translation, it is important to note that the application of targeted SERS NPs does not interfere with downstream IHC (see Figs 4 and 6), and that standard post-operative histopathology can still be performed on tissues after intraoperative QMP imaging. Consequently, QMP is not required to be a gold-standard diagnostic technique. Rather, the purpose of QMP is to greatly reduce the number of re-excision surgeries for breast cancer lumpectomy patients by helping surgeons to intraoperatively identify surgical margins that are positive for carcinoma in which there is an unequivocal need for additional resection. Conventional post-operative pathologic examination of tissue may still be performed as a gold standard to identify microscopic tumor burden or close margins (for institutions that continue to maintain a conservative criterion for re-excision).

Our studies with fresh tissues from animal models and human patients have shown that hemoglobin does not interfere with the acquisition and demultiplexing of topically applied SERS NPs. This is due to the high brightness of SERS NPs compared to intrinsic Raman scattering from blood or other tissue components. However, if necessary, reference Raman spectra can be included into demultiplexing algorithms to account for a variety of such background components. Another potential challenge is that cauterization of tissues may denature the cell-surface proteins targeted by SERS NPs. Note that cautery, used by many but not all surgeons when performing a lumpectomy, is also a challenge for conventional pathology, where it has been observed that certain protein targets and epitopes are more adversely affected than others by cautery^38^. A major advantage of QMP is the potential to perform multiplexed molecular analysis of a large panel of biomarkers (potentially 5 to 10) in order to mitigate the effects of cautery. Future studies will examine the effects of surgical cautery on the performance of intraoperative QMP.

Additional work is necessary to further improve the QMP technique. Brighter NPs would be of value to improve signal vs. background ratio, sensitivity and imaging speed. To promote the ease of intraoperative imaging, enhanced protocols and devices should be developed to hold, stain and rinse tissue specimens (e.g. biopsy shavings) obtained from the margins of a resection cavity. In addition, while a few studies have shown the feasibility of detecting and demultiplexing large panels (5–10) of nontargeted SERS NPs in animal models and *ex vivo* human tissues^15^,^39^, further work is needed to demonstrate the ability to quantify a large panel of disease-related biomarkers with targeted SERS NPs such that the QMP technique may accurately identify tumors in a variety of patients with heterogeneous biomarker expression patterns. These efforts will benefit from improvements in imaging hardware (e.g. spectrometer resolution, optical throughput, and fiber probe designs) as well as demultiplexing algorithms and the chemistry of the NPs themselves (e.g. binding avidity and brightness). Recently, we have also explored kinetic-modeling approaches to improve the quantification of QMP images, including the derivation of receptor binding potentials that correlate with the concentration of cell-surface receptors^37^. Similar approaches with systemically injected fluorescent probes have been shown to enable sensitive detection of microscopic tumor burden in lymph nodes^26^.

Finally, the QMP technique may potentially be combined with other imaging techniques, such as such as confocal mosaicing microscopy (CMM)^40^,^41^, light reflectance spectroscopy (LRS)^42^,^43^,^44^,^45^,^46^, autofluorescence lifetime measurement (AFLM)^43^, intrinsic Raman spectroscopy^47^,^48^,^49^, touch-prep cytology^50^,^51^, and intraoperative frozen-section pathology^7^,^52^. The alternative optical strategies mentioned above are in various stages of preclinical and/or clinical development, and all have the potential to improve lumpectomy procedures, but with certain limitations. For example, CMM is a method to obtain pathology-like microscopic images over large fields-of-view with freshly resected tissues, either in reflectance mode^40^,^41^ or in fluorescence mode through the topical application of passive or targeted fluorophores^53^,^54^,^55^. Spectroscopic methods (reflectance, Raman, autofluorescence lifetime) reveal the relative concentrations of basic chemical constituents such as hydrocarbons, lipids, nucleic acids, and/or tissue-scattering parameters, and have yielded some promising results for tumor detection in tissues but often with limited specificity^42^,^43^,^47^,^48^,^49^. Finally, studies on touch-prep cytology have been inconsistent in demonstrating value for the intraoperative assessment of breast cancer margins^50^,^51^ and the high fat content in breast tissues makes the preparation of frozen sections difficult during lumpectomy procedures^52^. Our technique offers information about macromolecular (cell-surface receptor) biomarkers that can complement the morphological information provided by CMM, and the chemical-bond/tissue scattering information provided by intrinsic tissue spectroscopy. The ability to image a panel of cell-surface biomarkers, which are known to play a fundamental role in tumor biology, will enable accurate identification of tumors in spite of molecular heterogeneity between patients, within patients over time, and even within single tumors^56^,^57^.

In summary, the QMP technology is capable of comprehensively imaging large biopsy shavings (>4 cm^2^) under time-constrained intraoperative conditions with sub-millimeter resolution for the detection of small residual tumors. QMP can quantify the expression of a multiplexed panel of well-known and well-characterized cell-surface biomarkers that are routinely used to accurately diagnose, stage, and personalize the treatment of cancer patients. Finally, QMP examines excised biopsy shavings that are commonly obtained, or could be obtained, at the final stages of a variety of tumor-resection procedures. *Ex vivo* imaging allows toxicity and sterility issues to be averted. Future studies will be required to assess the ability of intraoperative QMP imaging to accurately detect residual tumors at surgical margins in comparison to gold-standard post-operative pathology, with an ultimate goal of improving patient care by reducing the rate of re-excision surgeries associated with breast-cancer lumpectomy.

## Methods

### Raman imaging device and raster-scanning system

A miniature spectral-imaging probe has been developed to quantify the specific vs. nonspecific binding ratio of SERS NPs applied on tissue samples, and measurement linearity for concentrations in the range of 1–400 pM has been demonstrated for SERS NPs topically applied on glass slides, mouse tissues and rat esophagus (Supplementary Fig. S1 and previous publications^17^,^30^). As shown in Fig. 2, a low-power 785-nm diode laser (~10 mW at the tissue) is used to illuminate the tissue via a singlemode fiber, creating a laser spot with a diameter that can be tuned from 0.2 to 1 mm (imaging resolution) by changing the working distance between the probe tip and tissue surface from 2 to 6 mm. Raman-scattered photons from illuminated SERS NPs are collected by 36 multimode fibers and transmitted to a customized spectrometer (Andor Holospec), where they are dispersed onto a cooled deep-depletion spectroscopic CCD (Andor, Newton DU920P-BR-DD). The detector integration time in this study was 0.5 s.

For raster-scanning imaging, a two-axis translation stage was constructed (Newmark systems Inc., ET-50-11) as shown in Fig. 2. A two-axis stepper-motor controller (Newmark systems Inc., NSC-A2L) actuates the two stages under computer control through a custom LabVIEW program, allowing for a travel range of 50 × 50 mm^2^ with a tunable velocity of 1.3 μm/s to 20 mm/s. The raster-scanned imaging of an entire tissue sample was performed by fixing the imaging probe and scanning the tissue sample. The imaging probe was positioned at a 45-deg angle with respect to the tissue surface to minimize the collection of specular reflections.

### SERS NPs and functionalization

SERS NPs were purchased from Cabot Security Materials Inc. These NPs consist of a 60-nm-diameter gold core, a unique layer of Raman reporters adsorbed onto the surface of the gold cores, surrounded by a 60-nm-thick silica coating, resulting in an overall diameter of ~120 nm (Fig. 2c). Three “flavors” of NPs were used here, identified as S420, S421 and S440, each of which emits a characteristic Raman spectrum due to chemical differences in the Raman reporter layer (Fig. 2d, Supplementary Fig. S5). Additional details about the NPs are available in the literature^20^,^58^.

The SERS NPs were functionalized with monoclonal antibodies (mAb) to target the epidermal growth factor receptor (EGFR) or the human epidermal growth factor receptor 2 (HER2) as described in a previous conjugation protocol^17^. In addition, negative-control NPs were prepared by conjugating one flavor of NPs with an isotype control antibody (mouse IgG1). In brief, the NPs were first reacted with a fluorophore, Cyto 647-maleimide (Cytodiagnostics Inc, part No. NF647-3-01), for the purposes of flow-cytometry characterization experiments, and then conjugated with either an isotype control (Thermo Scientific, MA110407), an anti-EGFR (Thermo Scientific, MS-378-PABX), or an anti-HER2 (Thermo Scientific, MS-229-PABX) mAb at 500 molar equivalents per NP. The NP conjugates were stored at 4 °C and protected from light before use. UV-VIS spectrophotometry was used to measure the concentration of the NP conjugates.

### Cell culture and flow cytometry

The three cell lines employed in this study were U251 (Krackeler Scientific, 45-09063001), A431 (ATCC, CRL-1555) and SkBr3 (ATCC, HTB-30D). U251 and A431 cells were cultured in DMEM medium (Lonza, 12-604F) and SkBr3 cells were cultured in Mccoy’s 5A medium (Lonza, 12-688F), both of which were supplemented with 10% fetal bovine serum (FBS, Thermo Scientific, SH3008803) and 1% penicillin-streptomycin (Lonza, 17-602E). All cells were cultured at 37 °C with 5% CO_2_. Trypsin EDTA 1X (Mediatech, MT25051CI) was used to detach cells.

Flow cytometry samples were prepared by mixing 50-uL cell suspensions (0.2 million cells) with 50 μL of 100-pM NP conjugates for 15 min at room temperature protected from light under gentle agitation at 300 rpm, followed by three rounds of purification via centrifugation (400 g for 5 min) and supernatant-replacement (500 μL per rinse) with FACS buffer (20% FBS in PBS). Each cell line was split into equally sized samples which were individually stained by EGFR-NPs, HER2-NPs or isotype-NPs. In addition, one unstained cell sample was also analyzed.

### Mouse xenograft model and human breast tissues

Nude mice (Taconic Farms Inc, model NCRNU-F) were used to develop tumor xenografts. All animal experiments were performed in accordance with approved guidelines and all experimental protocols were approved by the Institutional Animal Care and Use Committee (IACUC) at Stony Brook University (#449417) and University of Washington IACUC (#4345-01). The cancer cells, A431 (1 × 10^6^), U251 (3 × 10^6^) and SkBr3 (5 × 10^6^), were individually suspended in matrigel (BD biosciences, 354234) at a 1:1 volume ratio to form a 200 μL mixture. At 7–9 weeks of age, nude mice were subcutaneously implanted by injecting the cell mixture at different sites on their flanks. A maximum of three sites were implanted on each mouse with a distance of ~2 cm between adjacent sites. After 3–5 weeks, when all tumors reached a size of 8 to 10 mm, the mice were euthanized by CO_2_ inhalation, followed by the surgical removal of implanted tumors as well as a few pieces of thigh muscle as normal controls. After imaging, the tissues were fixed with 10% formalin and submitted for histopathology (IHC and H&E staining).

Human breast tissue specimens were obtained and imaged within 1 to 2 hours after lumpectomy or mastectomy at the Memorial Sloan Kettering Cancer Center (MSKCC) or University of Washington Medical Center with patient consent. All experiments were carried out in accordance with approved guidelines and all experimental protocols were approved by the Institutional Review Board and Human Subjects Division at the University of Washington and the Northwest BioTrust (NWBT) under an IRB exemption for these de-identified tissues. After imaging, the tissues were fixed with 10% formalin and submitted for histopathology (IHC staining).

### Tissue staining and imaging

To optimize the staining and rinsing procedure, a step-by-step rinsing method was utilized. A 1-cm sized tissue sample was placed on a glass slide, and the top surface was stained with a 10-μL NP mixture, rinsed and imaged at a fixed 6-mm working distance (~1 mm illumination spot). Each rinse step was performed by gently spraying 100-μL of PBS onto the tissue surface and then removing any residual PBS. Raman spectra were acquired before rinsing (marked as Step 0 in Fig. 3a,b) and after each of ten rinse steps.

For raster scanning, tissue backgrounds were first obtained by imaging the entire tissue surface. The tissue surface was then stained with a mixture of 2 or 3 flavors of conjugated NPs (20–30 μL per 1 cm^2^ tissue area; 150 pM per flavor) with 1% BSA. After staining, the tissue sample was rinsed in 50-mL PBS with gentle agitation for 20 s, followed by raster-scanned imaging of the entire stained surface.

Raw NP spectra were demultiplexed to calculate the concentration and ratio of SERS NPs using a direct-classical-least-squares (DCLS) algorithm as described previously^17^. Other than sources of random noise, it was assumed that each measured spectrum consisted of a linear combination of individual NP spectra (obtained from pure NP flavors) and broadband background signals from tissues, buffers and glass substrates. Since the reference spectra of tissues can vary between sites due to differences in optical properties, a principal component analysis (PCA) was used, which has been shown to be a robust method to accommodate for variations in the background spectra^24^,^31^,^59^. In brief, before staining the human breast sample, the entire tissue was raster-scanned to obtain a complete set of background spectra (200–400 spectra depending on the tissue size). The acquired background spectra were then analyzed to calculate the first three principal components, as well as an average background spectrum, which were all used as background references and cumulatively could account for almost all of the observed background variations. After NP staining and imaging, the spectra acquired from stained tissues were demultiplexed using the DCLS algorithm to calculate the weight of each spectral component (e.g. the different flavors of NPs, the average tissue background, principal components of the tissue background, etc.). The NP concentrations were calculated based on calibration measurements with stock NPs of known concentrations.

### Statistical analysis

Statistical analysis was performed in Origin or Matlab. All values in the figures are presented as mean ± standard deviation unless otherwise noted in the text and figure captions. Statistical significance was calculated by a student’s t-test (two-sample, unpaired), and the level of significance was set at P < 0.001. For the box plots in Fig. 3d and Fig. 6b, the bottom and top of the box represent the 1st and 3rd quartiles of the dataset, respectively, and the band inside the box represents the median (2nd quartile) of the data.

## Additional Information

**How to cite this article**: Wang, Y. *et al.* Quantitative molecular phenotyping with topically applied SERS nanoparticles for intraoperative guidance of breast cancer lumpectomy. *Sci. Rep.* **6**, 21242; doi: 10.1038/srep21242 (2016).

## Supplementary Material

Supplementary Information

## Figures and Tables

**Figure 1:**
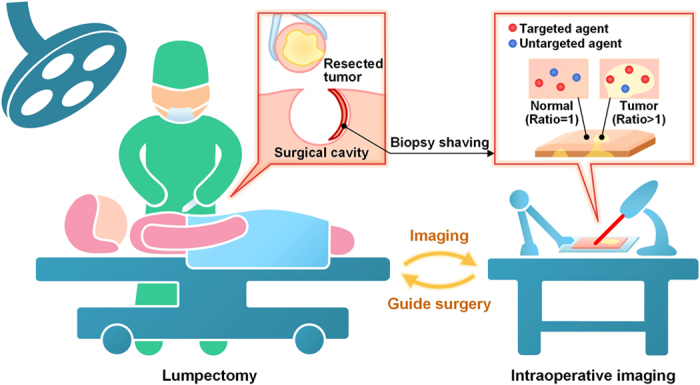
Schematic of an intraoperative imaging technique to rapidly identify residual tumors at the margins of freshly resected tissues for guiding breast-conserving surgeries. A ratiometric strategy (right inset) quantifies biomarker expression by comparing the signal from targeted NPs and nontargeted NPs. Y.W. drew the figure.
