# Supplementary material for: Quantitative molecular phenotyping with topically applied SERS nanoparticles for intraoperative guidance of breast cancer lumpectomy
Source: Sci Rep. 2016 Feb 16;6:21242. doi: 10.1038/srep21242 (PMC4754709; doi:10.1038/srep21242)
Supplement: Supplementary Information [file srep21242-s1.pdf]

Quantitative molecular phenotyping with topically applied SERS nanoparticles for intraoperative guidance of breast cancer lumpectomy

Yu "Winston" Wang,[1] Soyoung Kang,[1] Altaz Khan,[2] Gabriel Ruttner,[2] Steven Y. Leigh,[1,2] Melissa Murray,[3] Sanjee Abeytunge,[4] Gary Peterson,[4] Milind Rajadhyaksha,[4] Suzanne Dintzis,[5] Sara Javid,[6] and Jonathan T.C. Liu[1]*

[1] *Department of Mechanical Engineering, University of Washington, Seattle, WA 98195*
[2] *Department of Biomedical Engineering, Stony Brook University (SUNY), Stony Brook, NY 11794*
[3] *Department of Pathology, Memorial Sloan Kettering Cancer Center, New York, NY 10065*
[4] *Dermatology Service, Memorial Sloan Kettering Cancer Center, New York, NY 10065*
[5] *Department of Pathology, University of Washington School of Medicine, Seattle, WA 98195*
[6] *Department of Surgery, University of Washington School of Medicine, Seattle, WA 98195*
**jonliu@uw.edu*

Supplementary information

Linearity and limit-of-detection of spectral measurements

A linearity test was performed with SERS nanoparticle (NP) samples to demonstrate the accuracy of the imaging system. Two sets of NP samples were prepared with different mixture ratios. One set was prepared by mixing S420 and S440 in an equimolar ratio (1:1) and diluted to different concentrations (0.5, 1, 2.5, 5, 12.5, 25, 50, 100, 200 and 400 pM). The other set was prepared by mixing S420 and S440 in a 3:1 volume ratio and diluted to different concentrations (1.5, 3, 7.5, 15, 30, 75, 150, 300 and 600 pM for S420; 0.5, 1, 2.5, 5, 10, 25, 50, 100 and 200 pM for S440). A 1-μL drop from each sample was placed on a glass slide, and 3 acquisitions were taken with the imaging system by directing the laser beam through the center of the drop. The acquired spectra were then demultiplexed to calculate the NP weights. Since the same working distance (4 mm) was used for all the measurements, the concentration of NPs can be calculated based on a calibration measurement performed by recording the weights of known stock concentrations of S420 and S440. The measured concentrations (**Figs. S1a, S1c**) and concentration ratios of S420/S440 (**Figs. S1b, S1d**) both exhibit good linearity in the range of 1 to 400 pM, with larger errors (>20%) appearing below 1 pM. Therefore, all data presented in this paper correspond to NP concentrations considerably greater than 1 pM.

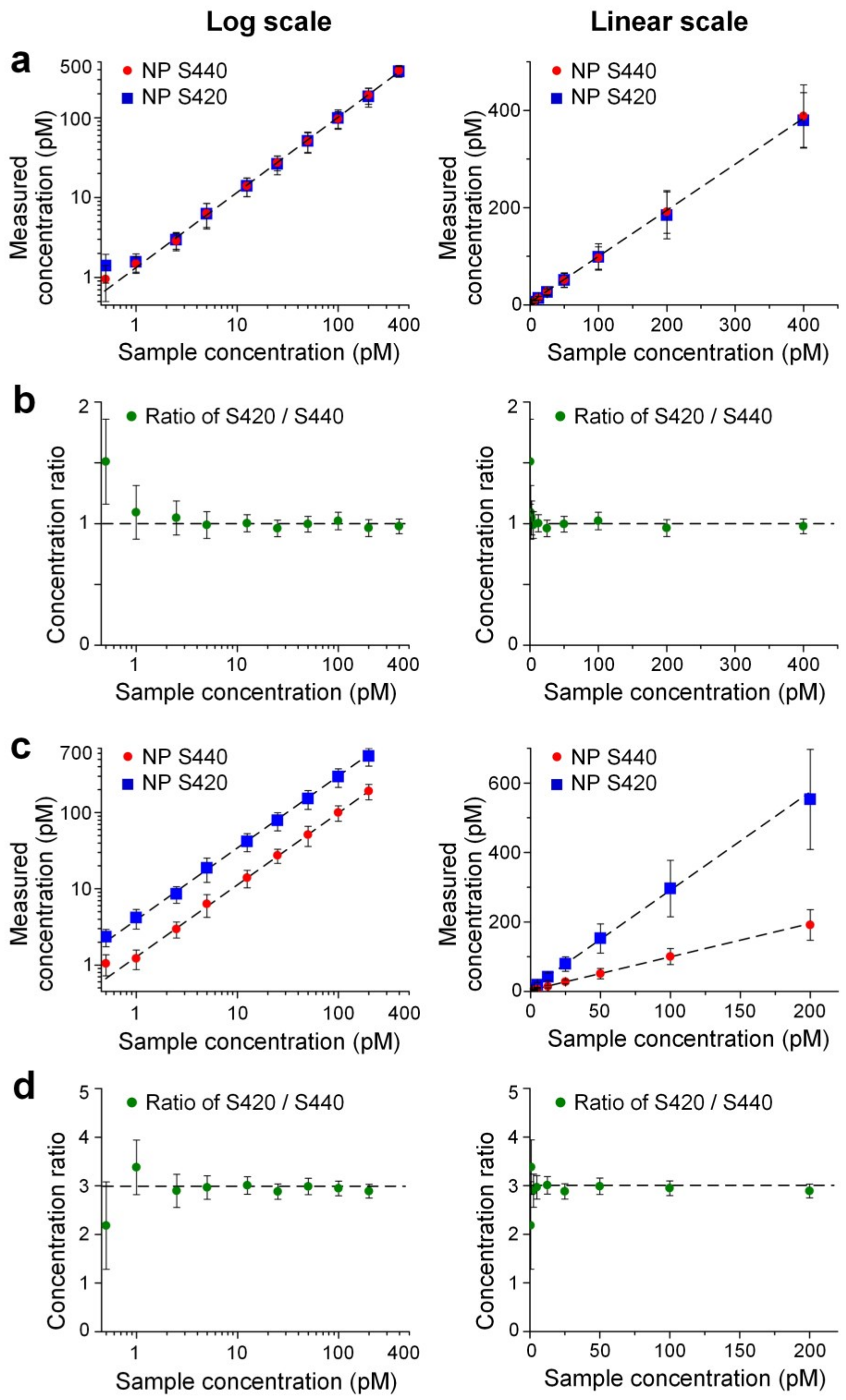

Figure S1. Linearity of spectral measurements. The NP flavors S420 and S440 were mixed in either a 1:1 ratio (**a, b**) or a 3:1 ratio (**c, d**) and diluted down from 400 pM to 0.5 pM. For each sample, three measurements were taken with the imaging system to calculate the NP concentrations (**a, c**) and concentration ratios (**b, d**). Error bars represent the standard deviation.

Specific-binding ability of mAb-conjugated NPs

Cultured cells were used to test the specific-binding ability of conjugated NPs via flow cytometry. Figure S2 shows the fluorescence histograms of a 10,000-cell analysis. The NPs show different binding levels between the cell samples by comparing the geometric mean of the fluorescent intensities (MFI). The MFIs of negative-control (biomarker-negative) 3T3 cells stained with the three NP conjugates (two targeted and one control) are similar. For EGFR detection, the binding level (MFI of EGFR-NPs vs. isotype-NPs) decreases according to the following order: A431 > U251 > SkBr3 > 3T3. For the HER2 detection, the binding level (MFI of HER2-NPs vs. isotype-NPs) decreases according to the following order: SkBr3 > A431 > U251 > 3T3. These results are consistent with the known receptor expression levels of these cell lines [1-5].

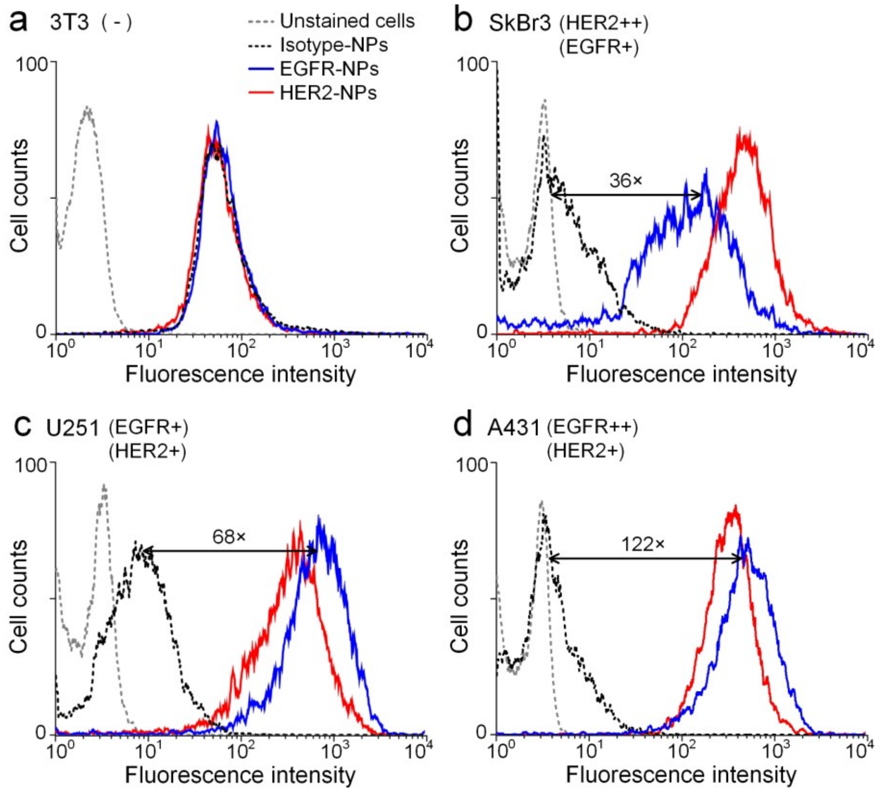

Figure S2. Flow cytometry validation of conjugated NPs with cultured cells. EGFR-NPs, HER2-NPs and isotype-NPs were individually used to stain (**a**) 3T3 (-), (**b**) SkBr3, (**c**) U251 and (**d**) A431 cell lines. Fluorescence histograms from unlabeled and NP-stained cells are shown.

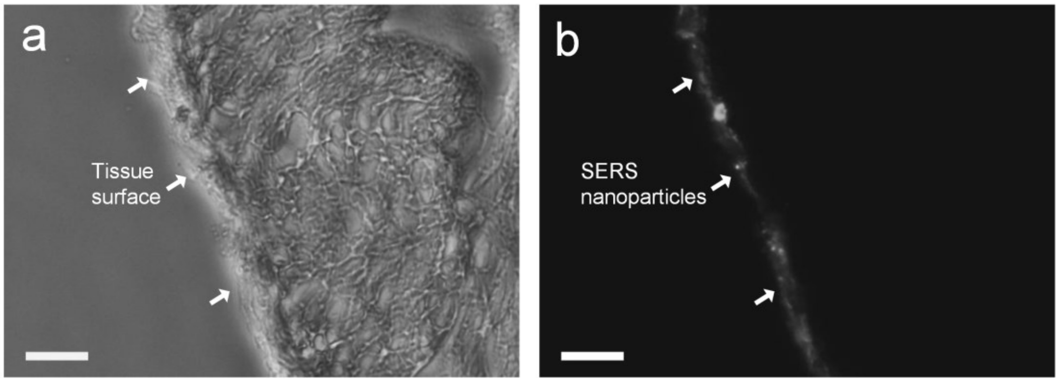

Figure S3. (**a**) Bright field and (**b**) fluorescence images showing that topically applied SERS NPs (conjugated to fluorophores in this case) are localized to the tissue surface. A piece of mouse muscle was topically stained with EGFR-NPs (300 pM, 10 min), rinsed in PBS (20 s) and embedded in O.C.T. (optimal cutting temperature) compound. A frozen section (10-μm thick) was cut and imaged with a fluorescence microscope. Scale bars represent 20 μm.

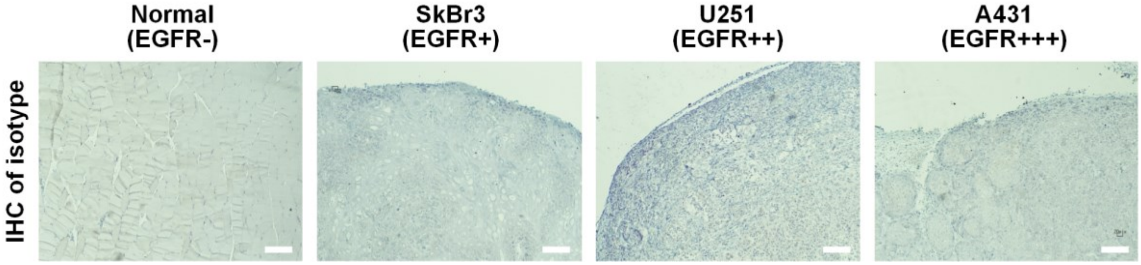

Figure S4. IHC validation images with mouse IgG1 isotype control antibody (supplementary data for Figure 4). Scale bars represent 100 μm.

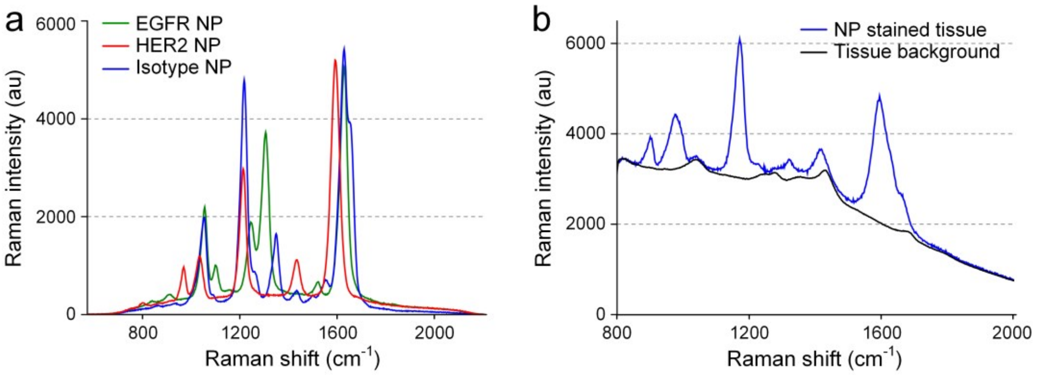

Figure S5. Raman spectra of (**a**) the three NP contrast agents used in this study and (**b**) NP-stained human breast tissues.

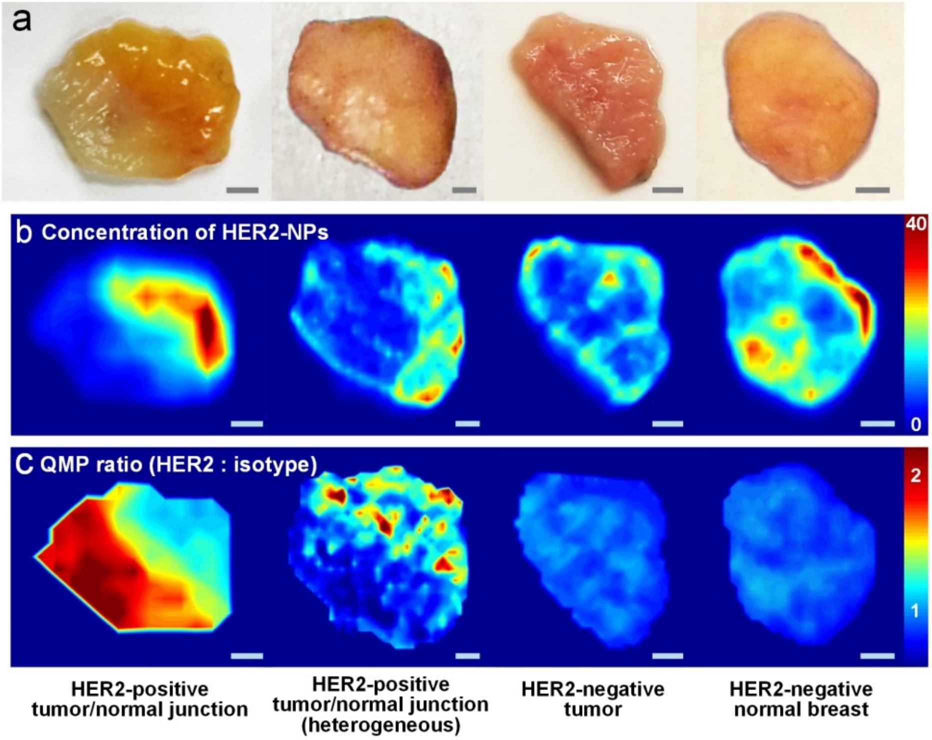

Figure S6. QMP imaging of human breast tissues stained with a 2-flavor NP mixture (HER2-NPs and isotype-NPs, 150 pM/flavor). (**a**) Photographs of four tissue specimens from four patients: two HER2-positive specimens containing both tumor and normal tissue regions and two HER2-negative specimens (one tumor and one normal tissue). Images showing (**b**) the absolute concentration of HER2-NPs, in which nonspecific accumulation of the NPs results in ambiguous results, and (**c**) the concentration ratio of HER2-NPs vs. isotype-NPs (resolution = 0.5 mm), which provides an accurate measure of specific vs. nonspecific NP retention in tissues that correlates with immunohistochemistry and flow cytometry. Scale bars represent 2 mm.

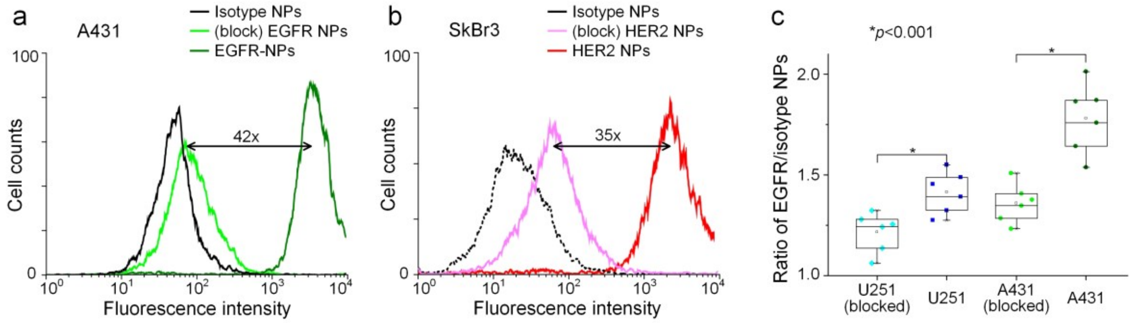

Figure S7. Flow cytometry and Raman imaging experiments with competitively inhibited cells/tumors. EGFR-NPs, HER2-NPs and isotype-NPs (100 pM) were individually used to stain (**a**) A431 and (**b**) SkBr3

cell lines (15 min). Fluorescence histograms are shown. Results show that when competitively inhibited with an excess of mAb, A431 cells (inhibited with anti-EGFR mAb and then stained with EGFR NPs) show a 42-fold reduction in mean fluorescence intensity (MFI), while SkBr3 cells (inhibited with anti-HER2 mAb and then stained with HER2 NPs) show a 35-fold reduction, suggesting that the EGFR-NPs and HER2-NPs bind specifically to cell-surface EGFR and HER2 receptors, respectively. (**c**) Concentration ratios of EGFR NPs vs. isotype control NPs acquired from U251 and A431 tumor xenografts implanted in mice. For each tumor type, two tumors were resected from two nude mice, and one tumor was blocked with 10 mM unlabeled anti-EGFR antibody for 2 hours. The blocked and untreated tumors were stained with an equimolar mixture of EGFR NPs and isotype control NPs (150 pM/flavor) for 10 min, followed by a 20-s rinse in PBS and Raman imaging. The competitively inhibited A431 and U251 tumors show decreased ratios, which suggest that the EGFR-NPs and HER2-NPs target EGFR and HER2 receptors, respectively. $^*p < 0.001$.

References

1. Schmidt, M., Hynes, N.E., Groner, B. & Wels, W. A bivalent single-chain antibody-toxin specific for ERBB-2 and the EGF receptor. *Int. J. Cancer* **65**, 538–546 (1996).
2. Wels, W. et al. EGF receptor and p185ERBB-2-specifi c single-chain antibody toxins differ in their cell-killing activity on tumor cells expressing both receptor proteins. *Int. J. Cancer* **60**, 137–144 (1995).
3. Gaborit, N. et al. Time-resolved fl uorescence resonance energy transfer (TR-FRET) to analyze the disruption of EGFR/HER2 dimers: A new method to evaluate the efficiency of targeted therapy using monoclonal antibodies. *J. Biol. Chem.* **286**, 11337–11345 (2011).
4. Habib, A.A., Chun, S.J., Neel, B.G. & Vartanian, T. Increased expression of epidermal growth factor receptor induces sequestration of extracellular signal-related kinases and selective attenuation of specifi c epidermal growth factor-mediated signal transduction pathways. *Mol. Cancer Res.* **1**, 219–233 (2003).
5. Moasser, M.M., Basso, A., Averbuch, S.D. & Rosen, N. The tyrosine kinase inhibitor ZD1839 ("iressa") inhibits HER2-driven signaling and suppresses the growth of HER2-overexpressing tumor cells. *Cancer Res.* **61**, 7184–7188 (2001).
